# From Glaciers to Refrigerators: the Population Genomics and Biocontrol Potential of the Black Yeast Aureobasidium subglaciale

**DOI:** 10.1128/spectrum.01455-22

**Published:** 2022-07-26

**Authors:** Janja Zajc, Anja Černoša, Xiaohuan Sun, Chao Fang, Nina Gunde-Cimerman, Zewei Song, Cene Gostinčar

**Affiliations:** a Plant Protection Department, Agricultural Institute of Slovenia, Ljubljana, Slovenia; b Department of Biology, Biotechnical Faculty, University of Ljubljana, Ljubljana, Slovenia; c BGI-Shenzhen, Shenzhen, China; d Lars Bolund Institute of Regenerative Medicine, BGI-Qingdao, Qingdao, China; University of Michigan

**Keywords:** cold adapted, genome analysis, antagonism, storage rot, apple decay

## Abstract

Apples are affected by numerous fungi known as storage rots, which cause significant losses before and after harvest. Concerns about increasing antimicrobial resistance, bans on various fungicides, and changing consumer preferences are motivating the search for safer means to prevent fruit rot. The use of antagonistic microbes has been shown to be an efficient and environmentally friendly alternative to conventional phytopharmaceuticals. Here, we investigate the potential of Aureobasidium subglaciale for postharvest rot control. We tested the antagonistic activity of 9 strains of *A. subglaciale* and 7 closely related strains against relevant phytopathogenic fungi under conditions simulating low-temperature storage: Botrytis cinerea, Penicillium expansum, and Colletotrichum acutatum. We also investigated a selection of phenotypic traits of all strains and sequenced their whole genomes. The tested strains significantly reduced postharvest rot of apples at low temperatures caused by *B. cinerea, C. acutatum* (over 60%), and *P. expansum* (about 40%). Several phenotypic traits were observed that may contribute to this biocontrol capacity: growth at low temperatures, tolerance to high temperatures and elevated solute concentrations, and strong production of several extracellular enzymes and siderophores. Population genomics revealed that 7 of the 15 strains originally identified as *A. subglaciale* most likely belong to other, possibly undescribed species of the same genus. In addition, the population structure and linkage disequilibrium of the species suggest that *A. subglaciale* is strictly clonal and therefore particularly well suited for use in biocontrol. Overall, these data suggest substantial biological control potential for *A. subglaciale*, which represents another promising biological agent for disease control in fresh fruit.

**IMPORTANCE** After harvest, fruits are often stored at low temperatures to prolong their life. However, despite the low temperatures, much of the fruit is lost to rot caused by a variety of fungi, resulting in major economic losses and food safety risks. An increasingly important environmentally friendly alternative to conventional methods of mitigating the effects of plant diseases is the use of microorganisms that act similarly to probiotics—occupying the available space, producing antimicrobial compounds, and consuming the nutrients needed by the rot-causing species. To find a new microorganism for biological control that is particularly suitable for cold storage of fruit, we tested different isolates of the cold-loving yeast *Aureobasidium subglaciale* and studied their phenotypic characteristics and genomes. We demonstrated that *A. subglaciale* can significantly reduce rotting of apples caused by three rot-causing molds at low temperatures and thus has great potential for preventing fruit rot during cold storage.

## INTRODUCTION

Aureobasidium subglaciale (Zalar, de Hoog & Gunde-Cimerman) Zalar, Gostinčar, Gunde-Cimerman (1) is a black yeast-like fungus notable for its narrow ecological amplitude and rare occurrence. Strains now classified as *A. subglaciale* had a status of variety within Aureobasidium pullulans (de Bary) Arnoud until 2014. *A. pullulans* shows remarkable adaptive abilities as well as a ubiquitous and abundant presence in temperate, polar, and tropical habitats. It is found in association with biotic substrates such as plants and animals and abiotic substrates indoors (house dust, household surfaces) and outdoors (rocks, soil, and aqueous environments). It is also found in extreme habitats such as hypersaline water of salterns, glacial ice, frozen or salt-preserved food, and even radiation-polluted areas (reviewed in Gostinčar et al. [1]). In contrast, the few *A. subglaciale* strains isolated so far have been limited to a narrow set of cold environments. The species was first found in the glacial ice of Spitsbergen glaciers (2), where the majority of currently known isolates were obtained. Later, an epiphytic strain from *Sphagnum* moss in Moscow (3), a strain from a radiation-polluted area of China (4), and several individual strains from Slovenia (three strains), Sweden (one), Netherlands (one), and Brazil and Argentina (two strains per each) were discovered (our unpublished data). The ecology of the existing *A. subglaciale* strains reflects their pronounced psychrophilic nature. So far, they have been found in either glacial or subglacial ice, in moss in the colder part of the year (3), or even in cool human-made environments, such as refrigerators (our unpublished data). In addition to its ability to grow at low ambient temperatures (4°C) (2), *A. subglaciale* also tolerates elevated salinity, high UV radiation, heavy metal contamination, and even gamma irradiation (4).

Despite such substantial differences in the ecological preferences of *A. subglaciale* and *A. pullulans*, the two species are not easily distinguishable in laboratory settings, due to their high phenotypic plasticity (1, 2, 5–7) and the resulting overlapping morphological and physiological traits of taxonomic importance (8). Although multilocus DNA sequence analysis distinguished between the two species and noted differences in temperature growth range, stress resistance, and degree of melanization (2), they were described as separate species only after the whole-genome sequencing revealed a large genomic distance between them (1).

Due to their phenotypic similarity, some of the biotechnological potential of *A. subglaciale* may be estimated from the many biotechnological uses of *A. pullulans*. The latter is known as a producer of pullulan, an extracellular polysaccharide used in cosmetics, medicine, and the food industry (9–11); numerous enzymes (amylases, lipases, and hemicellulose- and xylan-degrading enzymes) used in various industries (1, 6, 12, 13); and antifungal peptides (e.g., aureobasidin A) (14, 15). Besides pullulan production, the most commercially successful application of *A. pullulans* is in agriculture, where it is used as a biocontrol agent against several plant pathogens (16–18), with its effectiveness reported by numerous authors (reviewed in Zajc et al. [19]). Its use is expected to grow with increasing demand for fungicide-free environmentally friendly control of plant diseases.

Although preliminary, initial reports on the biocontrol potential of *A. subglaciale* suggested that the antagonistic activity of this species may be similar to that of *A. pullulans*. In two recent studies of the efficacy of different *Aureobasidium* strains against Botrytis cinerea (20, 21), *A. subglaciale* showed high efficacy in reducing pathogen growth with its soluble metabolites (21) as well as with its volatile organic compounds (20). These results indicated the need for further research of *A. subglaciale*, possibly leading to its commercial exploitation for biocontrol.

A major part of the motivation to study the biocontrol potential of *A. subglaciale* stems from its cold tolerance, which exceeds the cold tolerance of *A. pullulans*. Most fruits reach the market only after months of cold storage, during which, fungal decay often leads to significant losses. Apples are attacked by numerous fungi known as storage rots, which infect apples both in the orchards and after harvest. Besides wound-infecting (necrotrophic) pathogens such as Botrytis cinerea and Penicillium expansum, some important biotrophic pathogens (e.g., Colletotrichum acutatum and Colletotrichum gloeosporoides sp. complex) infect apples via lenticels or microcracks and cause latent infections later manifesting as storage rots (reviewed in Nybom et al. [22]). Fungal decay is especially problematic in the case of organic production, which is rising in importance due to consumer demand and due to the bans of numerous fungicides in many countries (22, 23). Therefore, alternative means of preventing postharvest decay are much needed, and antagonistic fungi have proven to be an effective tool to decrease the losses of fruit due to rot. Given the adaptation *of A. subglaciale* to low temperatures, its application as a biocontrol agent of produce stored under cool conditions appears particularly relevant.

To expand the repertoire of biocontrol *Aureobasidium* spp. with strains suitable for use at low temperatures used in cold storage of fruits, we investigated the antagonistic activity in all nine available strains of *A. subglaciale* and seven *A. subglaciale*-related strains previously identified as *A. subglaciale* (Table 1). To link the biocontrol potential of the strains with their phenotypic and genotypic traits, we performed the phenotypic characterization of all 16 strains and sequenced their whole genomes. We report on the relevant genomic and physiological traits contributing to the unique ecological fitness of *A. subglaciale* and to its promising antagonistic potential against *B. cinerea*, *P. expansum*, and *C. acutatum* (Table 2), three of the most problematic pre- and postharvest plant pathogens.

**TABLE 1:** List of *A. subglaciale* and related strains used in this study

Name in this study	Culture collection strain no.	Isolation habitat	Geographic location
A	EXF-2491	Subglacial ice collected from seawater	Arctic; Svalbard, Ny-Ålesund
B	EXF-2425	Subglacial ice collected from seawater	Arctic; Svalbard, Ny-Ålesund
C	EXF-2428	Subglacial ice collected from seawater	Arctic; Svalbard, Ny-Ålesund
D	EXF-2427	Subglacial ice collected from seawater	Arctic; Svalbard, Ny-Ålesund
E	EXF-2450	Glacier ice collected from sea water	Arctic; Svalbard, Ny-Ålesund
F	EXF-4632	Decaying plant leaves of *Convallaria* sp.	Slovenia
G	EXF-11962	Ice on inner household freezer wall	Slovenia; Golnik
H	EXF-12336	Refrigerator	Slovenia; Ljubljana
I^*a*^	EXF-8845	Lake water	Argentina; Puna
J^*a*^	EXF-8846	Lake water	Argentina; Puna
K^*a*^	EXF-10727	Integument of a male alate of Atta sexdens rubropilosa	Brazil; São Paulo, Botucatu
L^*a*^	EXF-10728	Integument of a male alate of *Atta sexdens rubropilosa*	Brazil; São Paulo, Botucatu
M^*a*^	EXF-12298	Refrigerator	Sweden
N^*a*^	EXF-12344	Refrigerator	Slovenia; Jezero
O^*a*^	EXF-3400	Meristematic clumps on window glass in a moist bathroom	Netherlands; Hilversum
R	EXF-2481; type strain; reference genome	Subglacial ice collected from seawater	Arctic; Svalbard, Ny-Ålesund

a *A. subglaciale-*related strains.

**TABLE 2:** List of phytopathogenic strains used in this study

Species	Culture collection strain no.	Isolation habitat	Geographic location
Botrytis cinerea	EXF-656	Chardonnay grapes	Slovenia; Drašiči
*Colletotrichum acutatum*	EXF-11123	Rotten apple	Slovenia; Ljubljana
*Penicillium expansum*	EXF-11121	Rotten apple	Slovenia; Ljubljana

## RESULTS

### Genomics.

**FIG 1:**
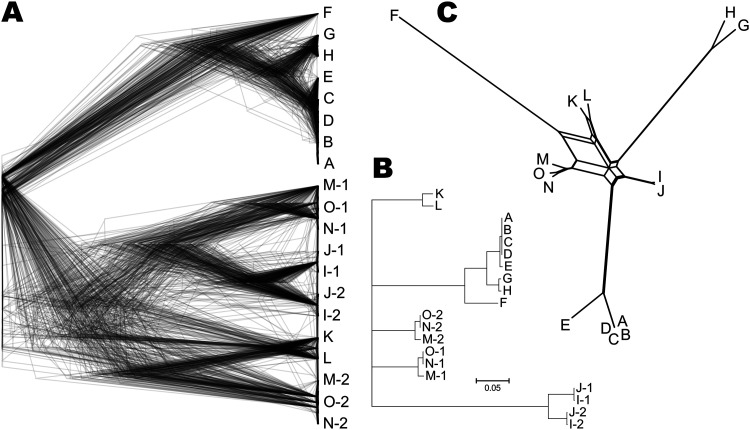
Phylogeny of the *Aureobasidium subglaciale* and related strains. The letters in the trees represent the source genomes (A to O) as named in this study. (A) Overlay of gene trees of 274 Benchmarking Universal Single Copy Orthologs (BUSCOs) estimated with PhyML 3.1 using the Hasegawa-Kishino-Yano 85 nucleotide substitution model and estimating the alpha parameter of the gamma distribution of the substitution rate categories and the proportion of invariable sites. Duplicate genes representing two haploid genomes of diploid strains are marked with the numbers 1 and 2. (B) Majority rule consensus tree of 274 core gene trees described above. (C) Phylogenetic network reconstructed using the Neighbor-Net algorithm based on the dissimilarity distance matrix calculated from the SNP data.

**FIG 2:**
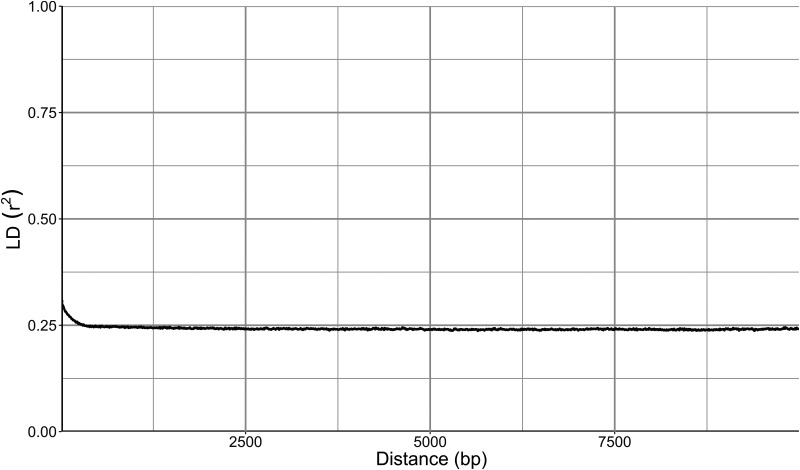
Linkage disequilibrium (LD) decay in *Aureobasidium subglaciale* and related strains estimated on all biallelic loci that were present in 25 to 75% of the sequenced genomes. LD measures were averaged in three nucleotide windows. Squared correlation coefficient (r*^2^*) between pairs of SNP loci plotted against the physical distance of the loci in the genome.

We examined the whole-genome sequences of 15 strains previously identified as *A. subglaciale* by phylogenetic analyses based on the internal transcribed spacer (ITS). One-third of the sequenced strains were diploid, namely, strains I, J, M, N, and O (Fig. 1A). The high congruence between the Benchmarking Universal Single-Copy Orthologs (BUSCO) gene phylogenies (Fig. 1A) indicated the lack of recombination between groups of strains. Clonality was further supported by the linkage disequilibrium squared correlation coefficient, which showed no decay even over large genomic distances (Fig. 2). A similar lack of decay of linkage disequilibrium was observed when only diploid genomes or only strains isolated from Svalbard were analyzed separately (data not shown), indicating a lack of recombination even in smaller or geographically close groups of strains.

The phylogenetic trees and phylogenetic network also showed a substantial phylogenetic distance between the core group of the *A. subglaciale* strains (A to E) originating from Svalbard glaciers and other strains (strains F to H; Fig. 1). Furthermore, the large phylogenetic distances suggested that strains I to O were initially misidentified based on sequencing of the ITS phylogenetic marker.

The average genome size of the sequenced *A. subglaciale* strains was 25.97 Mbp (standard deviation [SD]. ±0.54 Mbp) (Table 3), whereas the average genome size of the *A. subglaciale*-related strains was 43.80 Mbp (SD, ±11.93 Mbp), with the large average size attributable to diploid strains I, J, M, N, and O (see Table S1 in the supplemental material; Fig. 1). The average GC content of *A. subglaciale* strains was 50.66% (SD, ±0.08%), and that of *A. subglaciale*-related strains was 50.40% (SD, ±0.46%). The average number of predicted genes of *A. subglaciale* strains was 9,457 (SD, ±343), and the average gene length was 1,607 bp (SD, ±16 bp).

**TABLE 3:** Statistics for the *A. subglaciale* and related strains genomes sequenced in this study

Characteristic	Data for strain:							
	A	B	C	D	E	F	G	H
Genome assembly size (Mbp)	25.98	25.97	25.96	25.98	26.74	24.70	26.11	26.34
GC content (%)	50.73	50.73	50.73	50.73	50.69	50.56	50.57	50.55
CDS total length (Mbp)	14.35	14.38	14.38	14.38	14.19	13.27	13.77	13.89
CDS total length (% of genome)	55.23	55.37	55.39	55.37	53.04	53.74	52.74	52.73
No. of predicted genes	9,715	9,722	9,726	9,726	9,535	8,695	9,239	9,301
Avg gene length (bp)	1,594	1,597	1,596	1,596	1,607	1,645	1,608	1,610
No. of exons	25,214	25,294	25,253	25,266	24,940	22,567	24,007	24,203
Exons per gene (avg)	2.60	2.60	2.60	2.60	2.62	2.60	2.60	2.60
No. of introns	15,499	15,572	15,527	15,540	15,405	13,872	14,768	14,902
Avg intron length (bp)	75	76	75	75	76	76	75	75

**FIG 3:**
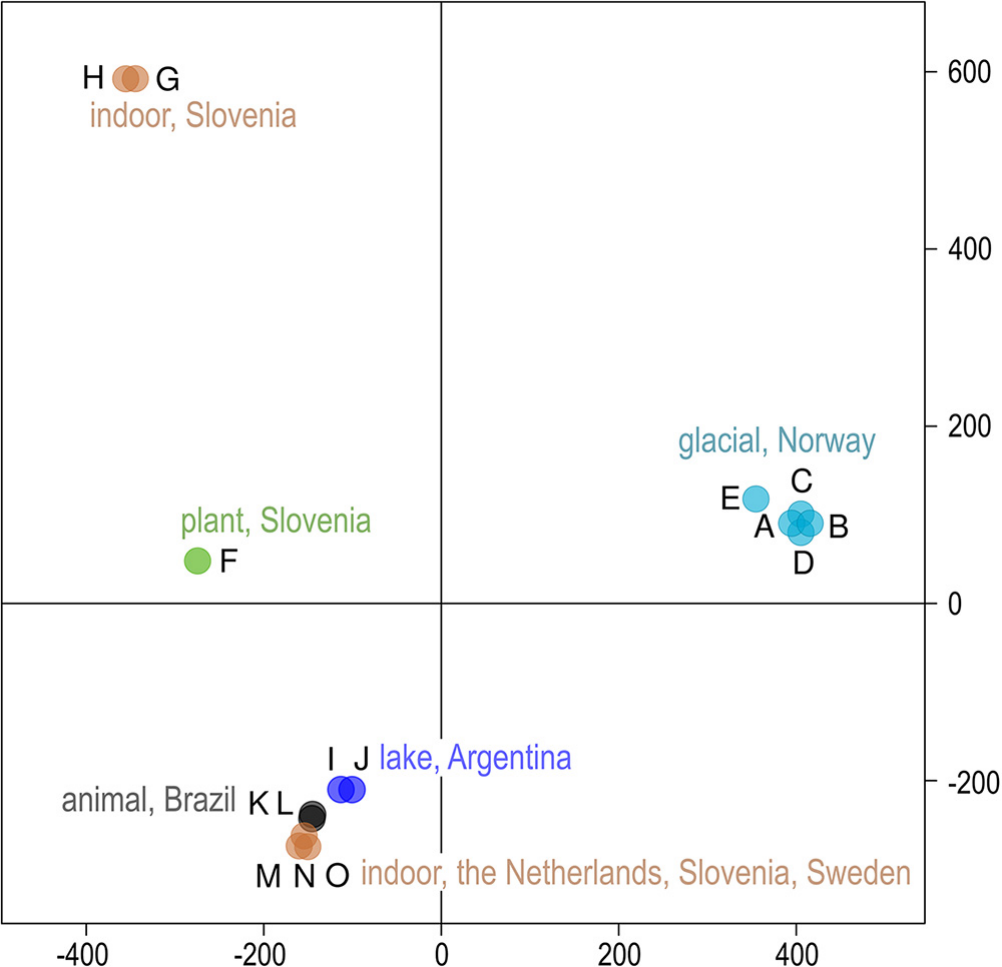
Clustering of *Aureobasidium subglaciale* and related genomes. PCA of SNP data estimated by comparing the sequenced *A. subglaciale* genomes to the reference genome. The genomes are represented by circles, the color of which corresponds to the habitat and sampling location of the sequenced strains. The first two axes explain 26.7% (horizontal) and 25.0% (vertical) of the variation.

Consistent with the lack of recombination among strains and large genomic distances, the population of *A. subglaciale* appeared relatively structured. In the principal-component analysis (PCA) of single nucleotide polymorphisms (SNPs), the genomes of *A. subglaciale* formed three clusters: a cluster of strains A to E, a cluster of strains G and H, and a cluster of strains I to O; strain F was positioned separately (Fig. 3). Clustering was associated with both habitat and sampling location.

### Growth at different temperatures.

Most strains of *A. subglaciale* and related strains tested here grew at 0°C, with the exception of strains I and O (Table 4). All strains grew at 24°C and 30°C. None of the strains grew at 37°C.

**TABLE 4:** Growth of *A. subglaciale* and related strains at various temperatures on YNB medium^*a*^

Strain name in this study	0°C	24°C	30°C	37°C
A	+	+	+	–
B	+	+	+	–
C	+	+	+	–
D	+	+	+	–
E	+	+	+	–
F	+	+	+	–
G	+	+	+	–
H	+	+	+	–
I	–	+	+	–
J	+	+	+	–
K	+	+	+	–
L	+	+	+	–
M	+	+	+	–
N	+	+	+	–
O	–	+	+	–
R	+	+	+	–

a +, Good growth; –, no growth.

### Tolerance to high temperatures.

As presented in Table 5, all strains survived and recovered after 2 h of incubation at 50°C, except strains A and G. More than half of the strains also survived 4 h of incubation at 50°C, but the survival after 6 h of incubation declined rapidly. Only two strains survived after 24 h of incubation at 50°C (strains M and N).

**TABLE 5:** Tolerance to high temperature (50°C) of *A. subglaciale* and related strains^*a*^

Name in this study	0 hours	2 hours	4 hours	6 hours	24 hours
A	+	–	–	–	–
B	+	+	+	+	–
C	+	+	–	–	–
D	+	+	+	+	–
E	+	+	+	–	–
F	+	+	+	+	–
G	+	–	–	–	–
H	+	+	–	–	–
I	+	+	–	–	–
J	+	+	–	–	–
K	+	+	+	+	–
L	+	+	+	–	–
M	+	+	+	+	+
N	+	+	+	+	+
O	+	+	–	–	–
R	+	+	+	–	–

a +, Good tolerance; –, no tolerance.

### Production of siderophores.

**FIG 4:**
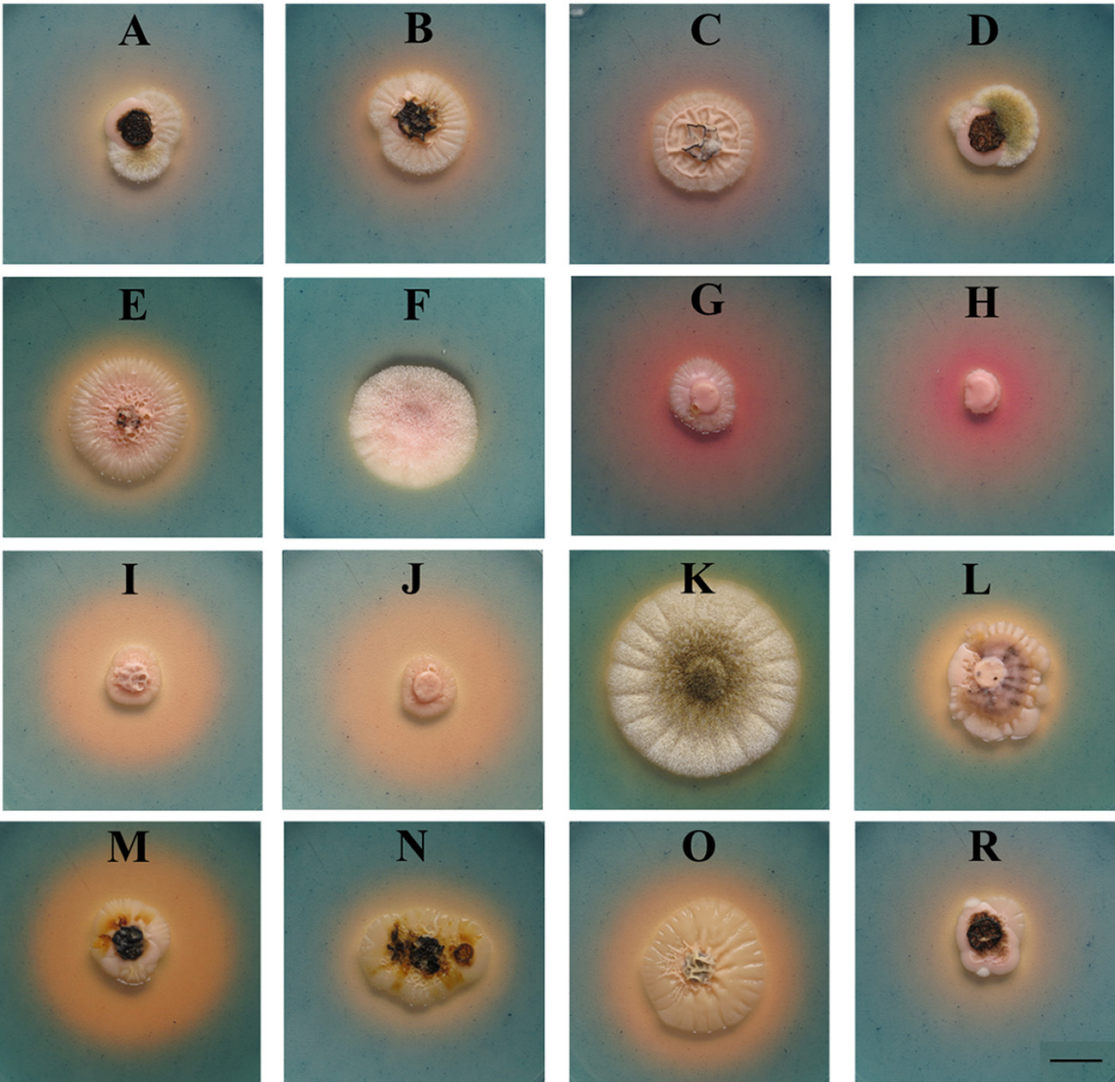
Siderophore production of *A. subglaciale* and related strains on CAS agar at 25°C. Strain letters (A to O, R) are listed in Table 1. Scale bar = 1 cm.

All strains produced siderophores, but the amount was variable. For instance, strains F and K showed only weak production, whereas strains G, I, J, M, and O showed strong production (Fig. 4).

Differently colored zones around colonies also indicate that different types of siderophores are produced. Catechol-type siderophores are indicated by the pink zones around the colonies (strains G and H), and hydroxamate-type siderophores are indicated by the yellow zones (all other tested strains) (19, 24).

We found genes for nonribosomal protein synthetases (NRPS) with adenylation domains (A domains) similar to those of SidC (responsible for the synthesis of triacetylfusarinin) and SidD (responsible for the synthesis of ferricrocin) in all sequenced genomes of *A. subglaciale* and related strains (Table 6). The majority of strains had two copies of the SidC homologue and three copies of the SidD homologue.

**TABLE 6:** Siderophore production and associated genes^*a*^

Strain name in this study	Relative amount of siderophores produced	No. of siderophores genes			
		SidC	SidD	FtrA	FetC
A	2.25	2	3	1	1
B	1.87	2	3	1	1
C	1.96	2	3	1	1
D	1.87	2	3	1	1
E	1.55	2	3	1	1
F	1.29	2	3	1	1
G	3.92	2	3	1	1
H	6.14	2	3	1	0
**I**	3.67	4	7	2	3
**J**	3.67	3.5	6.5	2	2.5
K	1.13	2	3	1	2
L	1.66	2	3	1	2
**M**	2.78	2.5	4	1	1
**N**	1.57	2	4	1	1
**O**	1.66	2	4	1	1
R	2.02	2	3	1	1

a The number of nonribosomal protein synthase (NRPS) genes with adenylation domains (A domains) similar to those of genes encoding proteins SidC and SidD and the number of homologues of FtrA/FetC complex identified in *A. subglaciale* and related genomes. Diploid strains are indicated by bold font, and the numbers of homologues are represented as per haploid genome.

Homologues of genes encoding the proteins of the FtrA/FetC complex were found in the genomes of all sequenced *A. subglaciale* and related strains except one (strain H). The majority of strains had one copy of each protein homologue.

### Enzymatic activities.

All strains showed strong amylase, esterase, chitinase and β-glucosidase activity (Table 7). A majority of strains also showed strong proteolytic, cellulolytic, and pectinolytic activities, with the exception of strain K (weak proteolytic activity), strains B and H (weak cellulolytic activity), and strains K, M, N, and O (weak pectinolytic activity). Xylanase production was strong in nine and weak in seven strains.

**TABLE 7:** Enzymatic activity of *A. subglaciale* and related strains^*a*^

Strain name in this study	Amylase	β-Glucosidase	Caseinase	Cellulase	Chitinase	Esterase	Pectinase	Xylanase
A	+	+	+	+	+	+	+	+
B	+	+	+	+/–	+	+	+	+
C	+	+	+	+	+	+	+	+
D	+	+	+	+	+	+	+	+
E	+	+	+	+	+	+	+	+
F	+	+	+	+	+	+	+	+/–
G	+	+	+	+	+	+	+	+/–
H	+	+	+	+/–	+	+	+	+/–
I	+	+	+	+	+	+	+	+
J	+	+	+	+	+	+	+	+
K	+	+	+/–	+	+	+	+/–	+/–
L	+	+	+	+	+	+	+	+
M	+	+	+	+	+	+	+/–	+
N	+	+	+	+	+	+	+/–	+/–
O	+	+	+	+	+	+	+/–	+/–
R	+	+	+	+	+	+	+	+/–

a +, Good activity; +/– weak activity; –, no activity.

**FIG 5:**
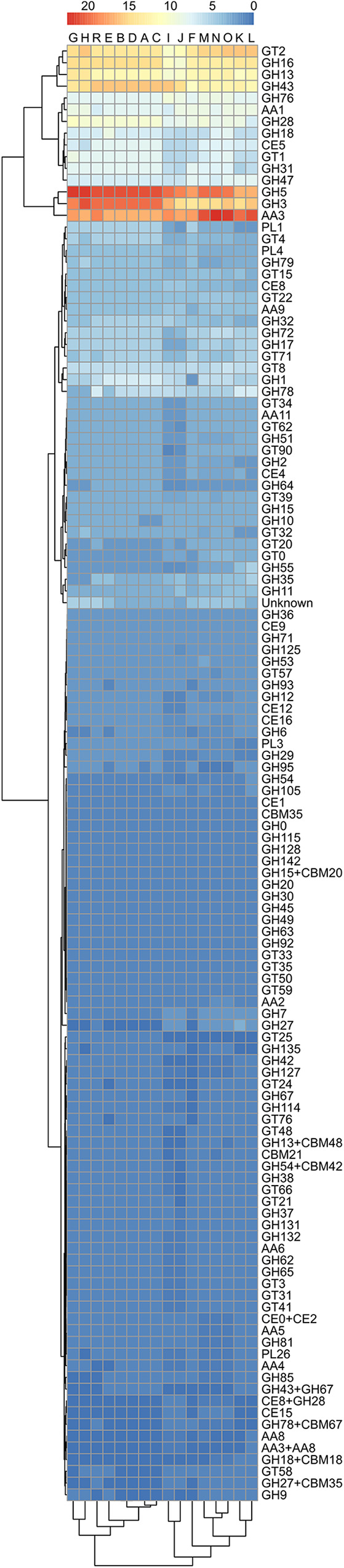
Predicted numbers of carbohydrate active enzymes (CAZymes) in the proteomes of *A. subglaciale* and related strains, according to the dbCAN server. The horizontal represents the tested strains and the vertical represents different CAZymes. The color codes correspond to the number of homologues. Dendrograms adjacent to the heatmap show the hierarchical clustering of the data.

The search for carbohydrate-active enzymes (CAZy) in the predicted proteomes of *A. subglaciale* and related strains (Fig. 5) led to the identification of many predicted proteins belonging to the CAZy families GH3, GH5, GH13, GH16, GH43 (all associated with plant and fungal cell wall degradation [25]), GT2 (synthesis of the cell wall and polymers [26]), and AA3 (various enzymes with the main function of catalyzing redox reactions [27]). There were fewer copies of homologues belonging to the families GH18, GH31, GH76 (all associated with plant and fungal cell wall degradation [25]), GH28 (pectin degradation [25]), GH47 (protein glycosylation [25]), CE5 (cutinase [25]), GT1 (family of glycosyltransferases [28]), and AA1 (family of multicopper oxidases [29]).

### Tolerance to solutes.

All *A. subglaciale* and *A. subglaciale*-related strains grew in the presence of different concentrations of sorbitol (9.1% and 18.2% [wt/vol]) and calcium chloride (CaCl_2_) (2%, 4%, 5%, and 10%). None of the strains tested grew on copper sulfate (CuSO_4_) or sodium carbonate (Na_2_CO_3_) (for both salts 0.25%, 0.5%, 0.75%, and 1% were tested).

### Dual culture test.

**FIG 6:**
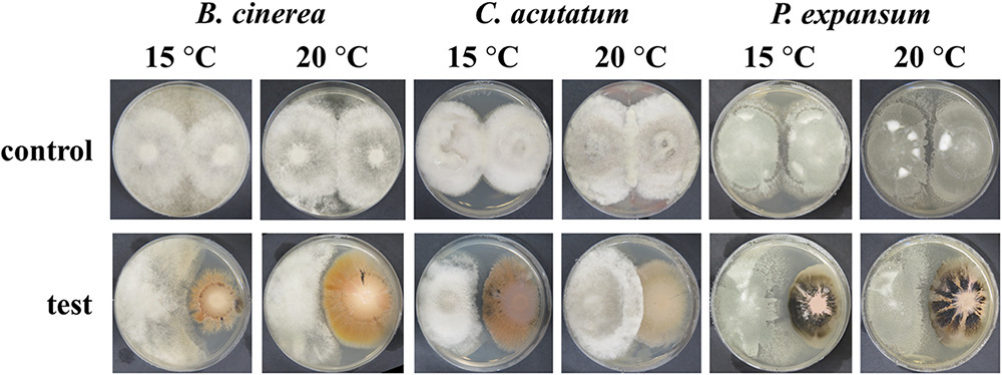
Representative images of the dual culture test. Plates were inoculated either with spores of phytopathogenic fungi only (control) or with spores of phytopathogenic fungi and cell suspension of biocontrol strains (test). Plate size, 70 mm.

We tested the activity of *A. subglaciale* and related strains on peptone-dextrose agar (PDA) plates by simultaneously growing both the biocontrol strain and the phytopatogenic fungi (Fig. 6). Inoculation of only pathogenic fungus served as a control.

Three-quarters of the *A. subglaciale* and related strains tested showed some activity against at least one phytopathogenic fungus at a minimum of one of the tested temperatures (Table 8). Seven strains inhibited the growth of *B. cinerea* and *C. acutatum* at 15°C. Five strains inhibited the growth of *B. cinerea* at 20°C. None of the strains showed any activity against *C. acutatum* at 20°C. Nine strains inhibited the growth of *P. expansum* at 15°C, and one strain (strain E) inhibited growth at 20°C.

**TABLE 8:** Inhibition of phytopathogenic fungi Botrytis cinerea (EXF-656), *Colletotrichum acutatum* (EXF-11123), and *Penicillium expansum* (EXF-11121) by individual strains of *A. subglaciale* (A to O, R) under different incubation temperatures^*a*^

Strain name in this study	*B. cinerea*		*C. acutatum*		*P. expansum*	
	15°C	20°C	15°C	20°C	15°C	20°C
A	+	+	+	–	+	–
B	+	+	–	–	+	–
C	+	–	–	–	+	–
D	–	+	+	–	+	–
E	+	+	+	–	+	+
F	–	–	+	–	–	–
G	+	–	+	–	+	–
H	+	–	+	–	–	–
I	–	–	–	–	–	–
J	–	–	–	–	+	–
K	–	–	–	–	–	–
L	–	–	–	–	+	–
M	–	–	–	–	–	–
N	–	–	–	–	+	–
O	–	–	–	–	–	–
R	+	+	+	–	–	–

a +, Zone of inhibition or/and the reduction of the growth; –, no zone of inhibition or/and the reduction of the growth.

### Antagonistic activity on apples.

**FIG 7:**
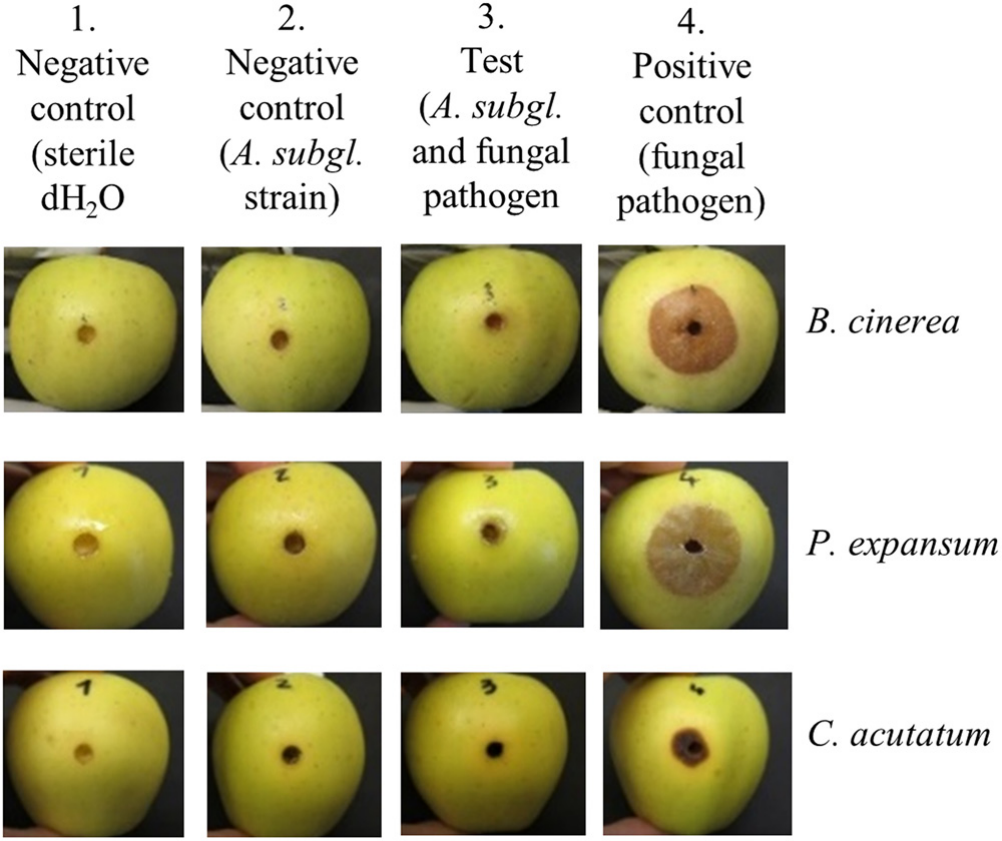
Representative images of the antagonism test of surface-sterilized golden delicious apples wounded and coinoculated with a spore suspension of a selected *A. subglaciale* strain and the pathogen (column 3) and incubated at 10°C. Each apple had its own controls—two negative controls, wound inoculated either with sterile water (column 1) or with an *A. subglaciale* strain (column 2), and a positive control (column 4) consisting of a wound infected with the pathogen alone. Apple size, 65 to 75 mm.

**FIG 8:**
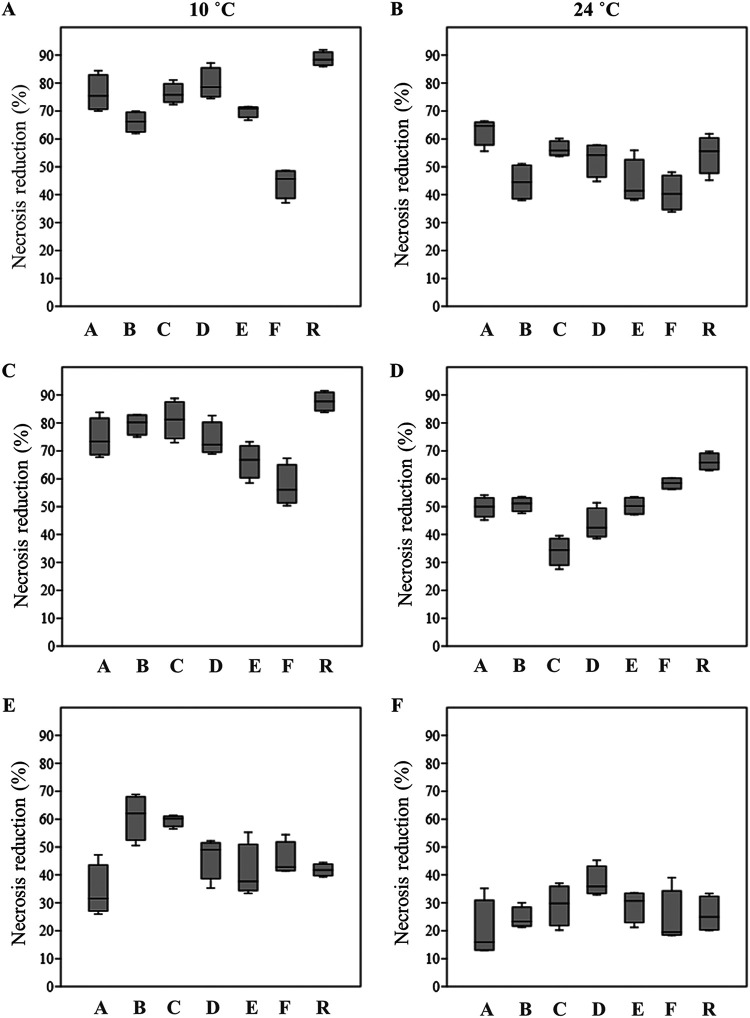
(A to F) Box plots showing the median (line in the box), 75th percentile and 25th percentile (upper and lower edges of the box), and minimum and maximum data values (whiskers) of *in vivo* antagonistic activity of *Aureobasidium subglaciale* and related biocontrol strains against *Botryis cinerea* (A, B), *Colletotrichum acutatum* (C, D), and *Penicillium expansum* (E, F) on ripe commercial golden delicious apples. Surface-sterilized apples were artificially wounded and inoculated with the combination of an individual biocontrol strain and the pathogen, including the positive (only the pathogen) and negative (sterile water and biocontrol strain) controls. After incubation at 10°C and 24°C, the necrosis reduction was determined by weighing the necrotic tissue and comparing it to the positive control.

Six *A. subglaciale* sensu stricto strains (A, B, C, D, E, and R) and one *A. subglaciale*-related strain (F) were selected for testing their antagonistic activity on apples against three postharvest pathogens, namely, *B. cinerea*, *C. acutatum*, and *P. expansum*. Six strains (A, B, C, D, E, and R) originated from subglacial ice (Arctic), and one strain (F) was isolated from decaying leaves of a *Convalaria* sp. plant (Slovenia). Ripe golden delicious apples were wounded on the equatorial line four times—two wounds served as negative controls (treated either with sterile water or *A. subglaciale* cell suspension), one wound served as the positive control (inoculated with the pathogen spore suspension), and one wound was the test of biocontrol potential of *A. subglaciale* evaluated as the ability to reduce necrosis of the wound due to the pathogen. In this way, each treatment of the individual apple had its own controls on the same fruit (Fig. 7). Our results clearly show that the strains of *A. subglaciale* exhibit substantial biocontrol potential against the three tested pathogenic fungi (Fig. 8) *in vivo*. All strains performed significantly (*P* < 0.05) better at 10°C than at 24°C against the three tested pathogens, except strain F, which showed comparable biocontrol activity against *B. cinerea* and *C. acutatum* at both tested temperatures (Fig. 8).

The strains of *A. subglaciale* showed significantly higher (*P* < 0.05) necrosis reduction of apple rot due to *B. cinerea* and *C. acutatum* infection than *P. expansum*. On average, the reduction of necrosis (Fig. 8) against *C. acutatum* and *B. cinerea* was comparable: 74.4% and 71.6% at 10°C; 50.4% and 50.8% at 24°C, respectively. In the case of wound infection with *P. expansum*, the reduction of necrosis of *A. subglaciale* was on average 47.0% and 27.1% at 10°C and 24°C, respectively.

All strains exhibited reduction of necrotic action of *B. cinerea*, which was for six of the strains greater than 60% and even reached 88% for one strain (R) at 10°C (Fig. 8A). At 24°C four strains showed necrosis inhibition greater than 50%, and one strain (A) showed inhibition greater than 60% (Fig. 8B). Similarly, all but strain 2 reduced necrosis due to infection with *C. acutatum* by 60%, even reaching 80% or higher (B, C, and R) (Fig. 8C and D). In the case of *P. expansum*, four strains reduced the necrosis by 40%, while two of them (B, C) reached 60% of inhibition at 10°C (Fig. 8E). At 24°C the inhibition of necrosis was lower than 40% (Fig. 8F).

## DISCUSSION

The use of *A. pullulans* as a biocontrol agent against various plant pathogens is increasingly commercially successful. However, biocontrol with microbial antagonists is a relatively new field, and its initial successes should be built upon by adding novel biocontrol agents. Especially interesting are specialized agents suitable for specific conditions or specific pathogens. Here, we studied whether the characteristic cold tolerance of *A. subglaciale*, a sister species of *A. pullulans*, qualifies this species as a useful biocontrol agent of fungal pathogens causing storage rot of fruits stored at low temperature.

*Aureobasidium subglaciale*, until 2014 recognized as a variety of *Aureobasidium pullulans*, is a rare species found in cold environments (2–4; our unpublished data) that can tolerate a variety of extreme conditions (1, 4). Preliminary studies of its biocontrol potential produced promising results (21). We examined the biocontrol-relevant phenotypic traits of all 16 currently available strains of *A. subglaciale* and sequenced 15 genomes to perform population genomic analyses and facilitate future studies of this species. We focused on the traits relevant to the application of this species as an antagonistic agent and tested the biocontrol performance of seven strains on apples in cold storage infected with some of the most problematic phytopathogenic fungi, Botrytis cinerea, *Penicillium expansum*, and *Colletotrichum acutatum.*

Due to the relatively recent description of the species and very sporadic isolation from nature, not much is known about *A. subglaciale*. Before this study only one whole genome of *A. subglaciale* (EXF-2481; R) had been sequenced and was found to be haploid and 25.80 Mbp in size (1). Two thirds of the strain sequenced here were also haploid and of similar size, although strains I, J, M, N, and O were diploid (Fig. 1A). This is not unprecedented in *Aureobasidium* spp.: while all *A. pullulans* isolates appear to be haploid (30), several diploid strains were found in *A. melanogenum* (31). Further investigation showed that strains I, J, M, N, and O may belong to other *Aureobasidium* species (Fig. 1A).

SNPs of *A. subglaciale* reflected a clear population structure of the species (Fig. 3), which corresponded to the habitat and geographic location from which the strains were isolated. To some extent this observation may be attributed to the apparent lack of recombination within the species, supported both by the strong phylogenetic signal seen in the congruence between phylogenies of different genes and also by the lack of decay of linkage disequilibrium even over large genomic distances (Fig. 2). Similar signs of clonality were found also in *A. melanogenum* (31), but not in *A. pullulans*, which shows signs of intense recombination (30). The reproductive strategy of the genus *Aureobasidium* thus appears to be species specific, a phenomenon that has been observed also in the genetically well-researched genus *Neurospora* (32). For the potential application of *A. subglaciale* strains as biocontrol agents, their clonality is certainly beneficial. On the one hand, it means that the strains will likely preserve their efficient genomic configuration; on the other hand, it reduces concerns about the potential negative outcomes of the recombination of the biocontrol agent with the naturally present wild strains of the same species (33). From this perspective, *A. subglaciale* may even be a more suitable biocontrol agent than *A. pullulans*.

Genomic population analyses have also shown that the resolution of fungal barcode phylogenetic marker ITS is not sufficient to fully distinguish between different *Aureobasidium* spp. Some strains included in this study as *A. subglaciale* may actually belong to other species. Based on intergenomic distances, strains M, N, and O are closer to *A. melanogenum* than to *A. subglaciale*, while strains I, J, K, and L possibly belong to a species new to science (Fig. S1). However, since their taxonomic status remains unresolved, they continue to be referred to as *A. subglaciale* in this study. The taxonomy of the genus *Aureobasidium* is complex (31), and its revision may be overdue, particularly in light of the high relevance of the genus in biotechnology. Our research presented here and past research suggest that comparative genomics could importantly contribute to such future taxonomic efforts.

Several phenotypic traits of *A. subglaciale* that are important for commercial biocontrol application were examined: growth at low temperatures, heat stress, tolerance to high concentrations of selected solutes, iron acquisition, and characterization of its enzymatic repertoire. It was previously determined that the optimal temperature of *A. subglaciale* is 25°C and that it can grow at 4 to 25°C (2). However, this study revealed that the temperature range of growth is wider than initially reported, as all the strains belonging to *A. subglaciale* were able to grow at 0 to 30°C. The cold tolerance of *A. subglaciale* makes this species ideally suited for use on produce stored in cold storage rooms at temperatures below 10°C. Equally important, none of them grew at human body temperature (37°C), which is one of the key virulence factors enabling fungi to infect mammals (34–36). This indicates that at least in terms of potential pathogenicity, *A. subglaciale* strains are safe to use in food production and manipulation. Interestingly, although unable to grow at 37°C, *A. subglaciale* strains showed good short-term survival at high temperatures. Almost all tested strains survived the 2-h temperature shock at 50°C, and some were even more resilient. This indicates substantial flexibility and stress tolerance of the species and presents an important trait in biocontrol, where robust biotic agents are preferred to ease their manipulation and application. For instance, unpredictable temperature fluctuations can occasionally occur during harvesting, manipulation, transportation, and storage of fruits and vegetables.

It was previously recognized that *A. subglaciale* is osmotolerant and can tolerate hypoosomotic or hyperosmotic shocks and can grow at elevated concentrations of salts or organic solutes (1, 2). Such a flexible response to osmotic shock is at least partially mediated via carefully regulated intracellular glycerol management (37) and extrusion of toxic cations (38). The cell suspensions of *A. subglaciale* strains were prepared in deionized water. The impact of this hypoosmotic shock was expected to be negligible, and robust tolerance of changing osmotic conditions is also beneficial in biocontrol applications. Of particular relevance are high concentrations of sorbitol and CaCl_2_. Sorbitol is a primary photosynthetic product (60 to 80%) in apples and many other tree fruits of the Rosaceae family and, interestingly, also plays an important role in plant-microbe interactions: it is a carbon source for microbes, but it also modulates plant defense responses to pathogens. Transgenic apples with decreased sorbitol levels are more susceptible to infections with Alternaria alternata, with exogenous application of sorbitol partially restoring resistance (39). Furthermore, it was recently published that sorbitol treatment of a biocontrol yeast, Debarymyces hansenii, improved its ability to inhibit *P. expansum* and *B. cinerea* infections (40). Here, we confirm a high tolerance of *A. subglaciale* to sorbitol: all tested strains grew at concentrations above 18%.

Calcium chloride treatment of fruit was shown to be effective in the control of postharvest rot. It can improve the activity of microbial antagonists against postharvest decay of a variety of fruits (41, 42). The tested strains of *A. subglaciale* were all able to grow on media supplemented with up to 10% CaCl_2_. This suggests that the biocontrol preparation of *A. subglaciale* could be applied together with CaCl_2_ solution to enhance the efficacy of postharvest rot control. Ippolito et al. (41) showed that the combination of *A. pullulans* (strain L47) with CaCl_2_ increased the level of protection against *Botrytis* rot on sweet cherries compared to treatments with either the yeast or chemical alone (41).

Iron is essential for the growth and development of all living organisms as a cofactor of enzymes and oxygen carrier in a variety of cellular functions (43). The ability to access the often-limited bioavailable iron in the environment represents an important competitive advantage and a way for biocontrol strains to outcompete pathogenic microorganisms. One of the most important mechanisms of iron acquisition is the production of siderophores, high-affinity iron-chelating compounds (44, 45). Siderophore-producing biocontrol strains are better equipped for outcompeting plant pathogens. All our *A. subglaciale* strains produced siderophores, but in different amounts and of different types—hydroxamate, indicated by the yellow color of the chrome azurol S (CAS) blue medium, and the catechol-type, indicated by the pink color (19, 24). Fungi typically produce hydroxamate siderophores, and only one example of fungal catecholate siderophores has been confirmed to date: pistillarin produced by Penicillium bilaii (46, 47). Therefore, the possible production of catecholate siderophores by some *A. subglaciale* strains warrants further investigation to complement studies of the mechanism and chemical properties of siderophores produced by the closely related *A. melanogenum*, which produces only hydroxamate siderophores (48–51). Despite variations in the amount and type of siderophores produced by the different strains of *A. subglaciale*, our *in silico* analyses showed no obvious differences in the number of gene copies encoding SidC and SidD, except for strains I and J (Table 6). This suggests that other mechanisms, rather than gene copy number, play a role in the expression of the siderophores.

The strains of *A. subglaciale* produced a similar repertoire of enzymes: amylases, esterases, chitinases, β-glucosidases, caseinases, cellulases, pectinases, and xylanases, although in some cases these enzymatic activities were weak (Table 7). Some of these enzymes, e.g., caseinases, β-glucosidases, chitinases, and cellulases, act directly on the phytopathogenic fungi and are therefore important in biocontrol applications (52–54). *In silico* analyses using the carbohydrate-active enzymes (CAZy) database showed that *A. subglaciale* strains contain genes belonging to a variety of CAZy families. The most abundant families were five families of glycoside hydrolases (GH5, GH3, GH16, GH13, and GH43) and one each of families belonging to glycosyltransferases, GT2, and auxiliary activities, AA3. The GH3, GH5, GH13, GH16, and GH43 families have been associated with the degradation of plant and fungal cell walls (25). Degradation of fungal cell wall is involved in direct parasitism—one of the mechanisms of action of antagonists against phytopathogenic fungi—and thus a desirable property of biocontrol agents. The CAZy AA3 family includes many different enzymes with different functions, the main feature of which is the catalysis of redox reactions. The enzymes of this family do not act directly on polymers such as cellulose, hemicellulose, pectin, and lignin, but they help other enzymes to depolymerize, e.g., lignocellulose (27). Enzymes from the GT2 family are involved in the synthesis of the cell wall (e.g., cellulose synthase) and various polymers (26).

To directly test the biocontrol performance of *A. subglaciale* strains, we performed two experiments: the dual culture test and the test of antagonism on postharvest apples. First, we tested the inhibition of the growth of the phytopathogenic fungi *B. cinerea*, *C. acutatum*, and *P. expansum* by *A. subglaciale* strains in the dual culture test at two different temperatures. Strains of *A. subglaciale* inhibited the growth of phytopathogenic fungi and did so more effectively at the low temperature. The *in vitro* antagonistic activity varied substantially between the strains, and some strains had no antagonistic activity in dual cultures (strains I, K, M, and O). Some strains of *A. subglaciale*, namely, A, D, E, and G, inhibited the growth of all three phytopathogenic fungi tested *in vitro*. The remaining strains showed antagonism only against some of the tested phytopathogens. This great variability could not be explained by the phenotypic and genotypic traits investigated in this study, which suggests that the *in vitro* antagonistic activity of *A. subglaciale* is additionally governed by as yet unidentified traits.

Based on their antagonism *in vitro*, and also based on the results of population genomics, we selected the best performing *A. subglaciale* sensu stricto strains for the study of antagonism *in vivo* and added strain F as the only strain isolated from the phyllosphere. Unlike the highly variable antagonistic activity of the wider selection of strains *in vitro*, the antagonistic activity of selected strains *in vivo* was much more consistent. All tested strains significantly reduced the necrosis of wounds inoculated with *B. cinerea*, *C. acutatum* and *P. expansum*, but to a variable degree – some strains performed significantly (*P* < 0.05) better than others against individual pathogens (Fig. 8). Similar to their performance *in vitro*, antagonism measured by the reduction of apple necrosis was generally better at a low temperature (10°C) compared to 24°C. An exception of this trend can be seen for strain F, which exhibited comparable biocontrol activity against *B. cinerea* and *C. acutatum* at both tested temperatures. The different performance of this strain may be linked to its large phylogenetic distance from other tested strains, as described above.

The discrepancy between the performance of biocontrol agents between *in vitro* tests such as dual culture plate assays and *in vivo* or *in planta* tests is a frequently observed phenomenon (20, 21) without a clear explanation. The conditions of the *in vitro* and *in vivo* tests are, of course, not comparable; there are numerous additional factors in *in vivo*/*in planta* trials, from the fruit biochemical contents to the complex interaction between the pathogen and its host (22). Plant defense mechanisms such as the production of reactive oxygen species (ROS) negatively affect the pathogen (and likely also the biocontrol species). Fungus, on the other hand, attempts to combat the plant’s innate immune system with the production of compounds such as phytotoxins, cell wall-degrading enzymes, and proteinaceous effectors (22), and these in turn lead to changes of the fruit composition (55, 56). As these interactions are specific to different combinations of the fungal pathogen and apple fruit cultivar (reviewed in Nybom et al. [22]), the biocontrol potential of *A. subglaciale* strains is also expected to be variable for the three pathogens tested here. For these reasons, *in vivo* and *in planta* experiments are essential. We believe that the data presented in this study, especially the encouraging *in vivo* antagonistic activity, are an important first step toward the potential acceptance of *A. subglaciale* as a commercial biocontrol agent of postharvest fruits, particularly during storage at low temperatures. The availability of genome sequences should facilitate further research into the mechanism of antagonistic activity of *A. subglaciale* via transcriptomic, metabolomic, and other approaches.

Based on the data presented here, we conclude that the black yeast *A. subglaciale* shows promise as a potential new biocontrol agent of fruit rot during cold storage. In this study, we showed significant reduction of postharvest necrosis of apples at low temperatures caused by *B. cinerea* EXF-656, *C. acutatum* EXF-11123 (over 60% reduction), and *P. expansum* EXF-11121 (approximately 40% reduction). In particular, strain R (EXF-2481) showed superior biocontrol performance against *B. cinerea* and *C. acutatum*, and strain B (EXF-2425) showed superior biocontrol performance against *P. expansum*. We also showed that many mechanisms of antagonistic activity of *A. subglaciale*, as well as of other fungi, remain unexplained. The genomic data produced by this study are expected to provide a good basis for future progress in all these fields: resolution of the complex taxonomy of *Aureobasidium*, biotechnological and biocontrol applications of *A. subglaciale*, and elucidation of its antagonistic activity against plant pathogens, contributing to reliable and environmentally friendly agriculture.

## MATERIALS AND METHODS

### Strains and growth conditions.

The strains of *A. subglaciale* and closely related strains (Table 1) were obtained from the Culture Collection Ex of Infrastructural Centre Mycosmo (Department of Biology, Biotechnical Faculty, University of Ljubljana, Slovenia). Strains were maintained on a defined yeast nitrogen base (YNB) medium (pH 7.0) consisting of 0.17% (wt/vol) yeast nitrogen base (Qbiogene, USA), 0.5% ammonium sulfate (Sigma-Aldrich, USA), 2% glucose (Kemika, Croatia), and 2% agar (Formedium, United Kingdom), dissolved in deionized water and sterilized by autoclaving.

For all experiments, except tolerance to high temperature, inoculums were prepared as cell suspensions in deionized water adjusted to an optical density at 600 nm (OD_600_) of 0.5. Strains K, N, and O grew in filamentous form; therefore, the medium was inoculated with 4-mm-diameter mycelial plugs cut from the margins of an actively growing colony.

The phytopathogenic strains were obtained from the Culture Collection Ex of Infrastructural Centre Mycosmo (Department of Biology, Biotechnical Faculty, University of Ljubljana, Slovenia). Strains were maintained on PDA medium.

### DNA extraction for genome sequencing.

Cultivation of the *Aureobasidium* sp. strains used in this study and DNA isolation were performed as previously described (34). Briefly, strains were grown at 24°C in defined liquid medium YNB on a rotary shaker at 180 rpm. Biomass was harvested in the middle of the exponential-growth phase (OD_600_, 0.8 to 1.0) by centrifugation (5,000 × *g* for 10 min), and cell pellets were frozen in liquid nitrogen and stored at −80°C until DNA isolation. The biomass was homogenized for 15 min with a pestle and mortar using liquid nitrogen. Then, 100 mg of the finely powdered biomass was placed in 2-mL microcentrifuge tubes with a Safe-Lock cap, containing a sterile stainless-steel ball. These tubes were placed in holders, precooled in liquid nitrogen, and shaken at a maximum speed (20 Hz) for 1 min (Retsch mixer mill 301; Thermo Fisher Scientific, USA) to achieve additional homogenization. The tubes were thawed on ice together with a 300-μL PowerBead solution (included in the UltraClean microbial DNA isolation kit; see below). These disrupted biomass suspensions were used for DNA extraction according to the manufacturer’s instructions using the UltraClean microbial DNA isolation kit (Mo Bio Laboratories, USA). RNA was removed using RNase A (Thermo Fisher Scientific, USA), and the quantity, purity, and integrity of the isolated DNA were evaluated using agarose electrophoresis, spectrophotometrically (NanoDrop 2000; Thermo Fisher Scientific, USA) and by fluorometry (Qubit; Thermo Fisher Scientific, USA).

### Genome sequencing.

Genome sequencing was performed on a DNA nanoball (DNB)-based platform T5 (DNBSEQ, MGI, China), with 2 × 100-bp libraries, prepared as previously described (57), in multiplex mode. The resulting output was demultiplexed, the quality was checked with FastQC, and the reads were trimmed for adaptors and quality (removal of bases with a Q value of <20) using the bbduk script (https://jgi.doe.gov/data-and-tools/bbtools/).

**Variant calling.** Sequencing reads were mapped to the reference *A. subglaciale* genome of strain EXF-2481 (GenBank AYYB00000000.1) (1) with the Burrows-Wheeler Aligner MEM algorithm (BWA-MEM), using the default parameters. The mapped reads were sorted using SAMtools 1.6 (58), and duplicates were marked with Picard 2.10.2. The density of the reference genome coverage by sequencing reads was calculated using SAMtools 1.6 (58) and visualized in R with ggplot2 (59, 60). Variant calling was performed with Genome Analysis Toolkit (GATK) 3.4 (61), following the (GATK) best practices workflow but using the “hard filtering” option.

**Assembly and annotation.** Genomes were assembled using IDBA-Hybrid 1.1.3 (62), using the genome of *A. subglaciale* EXF-2481 (1) as a reference to guide the assembly process. The maximum K value chosen was 100, the minimum support in each iteration was 2, the similarity for alignment was 0.95, the seed kmer was 20, the maximum allowed gap in the reference was 100, and the minimum size of contigs was 500 bp.

Annotation of protein-coding and tRNA genes was performed using MAKER 2.31.8 (63). The fungal subset of the Swiss-Prot database (recovered on 6 December 2018) and the published predicted proteomes of *A. pullulans*, *A. melanogenum*, *A. subglaciale*, and *A. namibiae* (1) were used as evidence. Two *ab initio* gene predictors were used in the MAKER pipeline. Semi-hidden Markov model (HMM)-based Nucleic Acid Parser (SNAP) (64) was bootstrap-trained within MAKER, based on the predicted genes derived from the alignment of the protein data sets to the genome, as recommended by Campbell et al. (63). AUGUSTUS was used with the training parameters for Neurospora crassa (65).

The completeness of the genome assembly and gene prediction was assessed with BUSCO 3 software (66) in proteomic mode, using the data set for ascomycetes (67). The default values were used for all of the parameters.

The files for submission to GenBank were prepared using Genome Annotation Generator (GAG) 2.0.1 software (68), removing all of the predicted genes with a coding DNA sequence (CDS) length of <150 bp or with introns of <10 bp.

**Variant-based analysis.** PCA of the SNP data was performed using the glPca function from the adgenet package (69). Linkage disequilibrium (LD) was estimated on a data set of biallelic SNP loci. For each pair of loci, the squared correlation coefficient (*r*^2^) was calculated using VCFtools (70). To examine LD decay, the *r*^2^ of loci within 10,000 nucleotides from each other was plotted as a function of distance (3-nucleotide window sliding average of all *r*^2^ was used to reduce noise) using ggplot2 in R (59, 60). A search for the LD decay range was performed, defined as the interval outside of which all of the arithmetic means of *r*^2^ were higher (left interval border) or lower (right interval border) than the maximum observed *r*^2^/2.

**Phylogenetic analysis.** Gene phylogenetic trees were constructed from the predicted coding sequences of all complete BUSCOs present in a single copy in haploid genomes and in two copies in diploid genomes. Sequences were aligned with MAFFT 7.407, with the –auto option and default parameters (71). This alignment was optimized using Gblocks 0.91, with the options -b3 = 10 -b4 = 3, and -b5 = n (72); if the resulting alignment was longer than 200 nucleotides and the average number of nucleotide differences between the sequence pairs was larger than 15 (as counted using the infoalign tool included in EMBOSS 6.6.0.0 [73]), the alignment was used for reconstruction of the phylogeny with PhyML 3.1 (74). The Hasegawa-Kishino-Yano 85 (75) nucleotide substitution model was used, with the alpha parameter of the gamma distribution of substitution rate categories and the proportion of invariable sites estimated using PhyML. The resulting trees were visualized using DensiTree 2.2.5 (76). A majority rule consensus tree was calculated with the consensus.edges function of the package phytools in R, using the default parameters (60, 77).

The phylogenetic network was reconstructed from the SNP data. The dissimilarity distance matrix was calculated using the R package poppr (78), and was used to construct the phylogenetic network with the Neighbor-Net algorithm, as implemented in the R package phangorn (60, 79).

Genomic distances between the sequenced genomes were calculated as recommended by Gostinčar (80).

**Identification of individual genes.** In the predicted proteomes of all sequenced *A. subglaciale* strains we identified the homologues of two nonribosomal peptide synthetases (SidC and SidD) responsible for the synthesis of siderophores. As the query, we used the previously identified adenylation domain (A-domain) of *A. subglaciale* (34) and performed the search with blastp and E value cutoffs of 10^−80^, 10^−40^, 10^−20^, and 10^−10^ according to Zajc et al. (35). In the same way, we searched for homologues of genes encoding the proteins of the FtrA/FetC complex, also with an E value cutoff of 10^−10^ (81, 82). For all matching proteins, we conducted a BLAST search against the GenBank nonredundant protein database to identify true homologues based on the annotation of similar GenBank proteins.

CAZys were identified in the predicted proteomes of *A. subglaciale* using a standalone instance of the dbCAN server (https://bcb.unl.edu/dbCAN2/index.php). Proteins were considered a CAZy if they were identified as such by at least two of the three tools used (i.e., HMMER, DIAMOND, and Hotpep). Visualization of the CAZy numbers was performed using the corrplot package in R (83).

### Growth at different temperatures.

We tested the growth of selected strains at 0°C, 24°C (growth optimum), 30°C, and 37°C (human body temperature) by spotting 5 µL of cell suspensions or adding one mycelial plug to YNB agar medium. Plates were incubated for 18 days at 0°C, for 8 days at 24°C, and for 10 days at 30°C and 37°C before growth evaluation with visual examination.

### Tolerance to high temperatures.

High-temperature tolerance was tested as previously described by Zajc et al. (19). Briefly, we prepared cell suspensions of the strains in a liquid YNB medium and adjusted them to an OD_600_ of 0.5 with a final volume of 1 mL. In the case of the filamentous form, we added 5 mycelial plugs (4 mm) excised from the margins of the growing colony to 1 mL of liquid YNB medium. The samples were then incubated at 50°C for 0, 2, 4, 6, and 24 h, respectively. At each time point, 100 µL of the sample was serially diluted to 10^−4^ in sterile deionized water in 10-fold steps, and 5 µL of each dilution was spotted onto a YNB agar plate. For filamentous fungi, one mycelial plug was put on YNB agar plates. The plates were incubated at 24°C for 10 days, after which colony growth was determined.

### Production of siderophores.

Production of siderophores was determined by plate assay using chrome azurol S (CAS) (44, 84) as previously described by Zajc et al. (19, 35). CAS agar was prepared as follows: first, the mixture of 10 mL of 1 mM FeCl_3_ × 6H_2_O (Sigma-Aldrich, USA) in 10 mM HCl (Merck, Germany), 50 mL of CAS solution (Acros Organics, USA), and 40 mL hexadecyltrimethylammonium bromide (CTAB) (Sigma-Aldrich, USA) was prepared. Then, the medium of 30.24 g piperazine-*N*,*N*′-bis(2-ethanesulfonic acid) (PIPES) (Acros Organics, USA), 12 g 50% (wt/wt) NaOH (Sigma-Aldrich, USA), 20 g malt extract (Biolife, Italy), 1 g peptone (Merck, Germany), 20 g glucose, and 20 g agar in 900 mL deionized water was prepared. Both solutions were autoclaved separately and carefully combined after cooling. Plates were inoculated with 5 µL of the cell suspension or with one mycelial plug and incubated at 25°C for 14 days. After the incubation, yellow, orange, or pink discoloration around the colonies was observed and regarded as production of siderophores.

The relative amount of produced siderophore was calculated according to Zajc et al. (35):

$$\text{Amount of siderophore produced }=\;\left(\text{diameter of colony and discoloration zone}\right)\times {\left(\text{diameter of colony}\right)}^{-\text{1}}$$

### Enzymatic activities.

The production of the various enzymes was determined by plate assays as previously described, modified according to Zajc et al. (19, 35). Briefly, amylolytic activity was determined on starch agar, and a positive reaction was observed as a clear zone around the colonies (85). The β-glucosidase activity was determined on esculin agar, and the positive reaction was observed as a black complex around the colonies (86). Proteolytic activity was determined using casein as a substrate. A positive reaction was defined as a clear zone around the colonies (87, 88). Cellulolytic activity was determined using carboxymethyl cellulose (CMC; Sigma-Aldrich, USA), and a clear zone around the colonies was considered a positive reaction (86). Chitinase activity was determined on medium containing colloidal chitin (from crab shells; Sigma-Aldrich, USA). A positive reaction was observed as a purple zone around the colonies (89). Esterase activity was determined on esterase medium, and a positive reaction was observed as a purple zone with white precipitation around the colonies (90). Pectinase activity was determined using apple pectin (Sigma-Aldrich, USA) as the substrate. A positive reaction was defined as a clear zone around the colonies (91, 92). Xylanase activity was determined on xylan medium, and a clear zone around the colonies was considered a positive reaction (86).

For all enzymatic tests, we spotted 5 µL of the cell suspension or put one mycelial plug onto suitable solid media in petri dishes and incubated them at 25°C for 7 days, except for the esterase assay plates, which were incubated for 5 days, and then examined the reactions on the plates.

### Tolerance to solutes.

To determine tolerance to solutes, we spotted 5 µL of cell suspension or put one mycelial plug on YNB agar plates supplemented either with sorbitol (9.1% and 18.2% [wt/vol]), CaCl_2_ (3%, 4%, 5% and 10% [wt/vol]), or CuSO_4_ (0.25%, 0.5%, 0.75% and 1% [wt/vol]). YNB medium without added solutes was used as a control. All plates were incubated at 25°C for 8 days.

### Dual culture test.

For the dual culture test, cell suspensions of *Aureobasidium* sp. strains were prepared in spore suspension solution (SSS) to an OD_600_ between 1.0 and 1.5. We spotted 10 µL of the cell suspension or placed one mycelial plug 1.5 cm from the edge of the PDA plate. We incubated plates at 25°C for 7 days. After incubation, we prepared spore suspensions of three phytopathogenic fungi (Botrytis cinerea EXF-656, *Colletotrichum acutatum* EXF-11123, and *Penicillium expansum* EXF-11121; Table 8). Spore suspensions were prepared in SSS at the concentration of 5 × 10^5^ spores/mL. Then, 10 μL of spore suspension was spotted onto a PDA plate 4 cm away from an *A. subglaciale* colony. As a control, 10 µL of spore suspensions of the same phytopathogenic fungi was spotted 4 cm apart without inoculation of *A. subglaciale* strains. We inoculated two plates for each condition (two biological replicates). Plates with *B. cinerea* were incubated at 15°C for 14 days and at 20°C for 7 days. Plates with *C. acutatum* were incubated at 15°C for 21 days and at 20°C for 14 days. Plates with *P. expansum* were incubated at 15°C and 20°C for 28 days. After incubation, we observed a zone of inhibition between the *A. subglaciale* strain and the phytopathogenic strain and/or the reduction of the growth of the phytopathogenic strain.

### Antagonism on apples.

A 1-µL inoculation loop of cultures of selected *A. subglaciale* (strains A, B, C, D, E, R) or a related strain (F) grown on PDA for 5 to 7 days at 24°C was resuspended in 10 mL of liquid potato dextrose medium and incubated without shaking overnight at 24°C. The cell suspension was centrifuged for 5 min at 5,000 × *g* at room temperature; the pellet was first washed and then resuspended in 10 mL sterile deionized water. The cell count was determined using the hemocytometer and adjusted to 10^7^ cells/mL.

The spore suspensions of the pathogen were prepared as follows: *B. cinerea* (EXF-656), *P. expansum* (EXF-11121), and *C. acutatum* (EXF-11123) were grown at 24°C on oatmeal agar (Difco, USA) for 7 to 14 days until sporulation occurred. The spore suspension was prepared in sterile 0.05% Tween 80. The spores were counted using the hemocytometer and adjusted to 5 × 10^4^ spores/mL.

Ripe commercial apples (golden delicious, 65 to 75 mm) were surface sterilized with 5% H_2_O_2_, rinsed with sterile deionized water, and dried under aseptic conditions. The apples were wounded 5 mm deep four times in the equatorial area with a cork borer (diameter, 5 mm). (i) The first wound was treated with 20 μL of sterile deionized water (negative control; no microorganism). (ii) The second wound was inoculated with 20 μL of 10^7^ cells/mL of an *A. subglaciale* strain (negative control; biocontrol agent only). (iii) The third wound was treated first with 20 μL of 10^7^ cells/mL of *A. subglaciale* or a related strain and after 2 h with 20 μL of pathogen suspension of 5 × 10^4^ spores/mL. (iv) The fourth wound (positive control; pathogen only) was treated with the pathogen suspension (20 μL, 5 × 10^4^ spores/mL).

The apples were individually packed into plastic bags and kept in the dark at 10°C and 24°C in an incubation chamber until necrosis developed in the wounds infected with the pathogen. After incubation, the necrotic lesions around the wounds were hollowed out with a 1-μL laboratory spatula and weighed. The experiment was performed twice; four apples were used per treatment. The negative-control wounds (i and ii) were without symptoms of necrosis. For wounds with combined activity of the *A. subglaciale* or related strains and the pathogen (iii), the necrosis reduction (NR) was calculated as follows:

$$\text{NR }=\;\left({\text{NW}}_{\text{4}}-{\text{NW}}_{\text{3}}\right)\;\times \;{\left({\text{NW}}_{\text{4}}\right)}^{-\text{1}}\times \text{ 1}00$$

where NW_3_ is the necrosis weight of the wound with the pathogen and *A. subglaciale* (in grams), and NW_4_ is the necrosis weight of the wound with the pathogen (in grams).

The results are presented as box plots with a median value (line) and minimal and maximal values of two independent experiments. One-way analysis of variance (ANOVA) and Tukey’s *post hoc* pairwise test were performed using PAST 3.20 software (93).

### Data availability.

The sequencing reads, assembly, and annotation data were deposited in GenBank under the BioProject accession number PRJNA527935. The data sets generated for this study can also be found in the China National GeneBank Sequence Archive (CNSA) (https://db.cngb.org/cnsa/), under the accession number CNP0000446.
