# Supplementary material for: From Glaciers to Refrigerators: the Population Genomics and Biocontrol Potential of the Black Yeast Aureobasidium subglaciale
Source: Microbiol Spectr. 2022 Jul 26;10(4):e01455-22. doi: 10.1128/spectrum.01455-22 (PMC9430960; doi:10.1128/spectrum.01455-22)
Supplement: Supplemental file 1 — Supplemental material. Download spectrum.01455-22-s0001.pdf, PDF file, 0.1 MB [file spectrum.01455-22-s0001.pdf]

Table S1: Statistics for the sequenced *A. subglaciale*-related genomes.

	I	J	K	L	M	N	O
**Genome assembly size (Mb)**	47,65	47,45	25,42	25,39	52,91	52,46	55,34
**GC content (%)**	49,96	49,96	51,11	51,10	50,29	50,20	50,18
**CDS total lenght (Mb)**	18,15	17,96	13,15	13,15	25,98	25,31	26,45
**CDS total lenght (% of genome)**	38,09	37,86	51,72	51,80	49,09	48,24	47,79
**Predicted genes (n)**	15073	14923	8477	8451	16948	16484	17264
**Gene average lenght (bp)**	1294	1295	1670	1677	1652	1653	1650
**Number of exons (n)**	34285	34061	22220	22111	44194	42951	44981
**Exons per gene (average)**	2,27	2,28	2,62	2,62	2,61	2,61	2,61
**Number of intron (n)**	19212	19138	13743	13660	27246	26467	27717
**Intron average lenght (bp)**	72	72	76	77	76	75	75

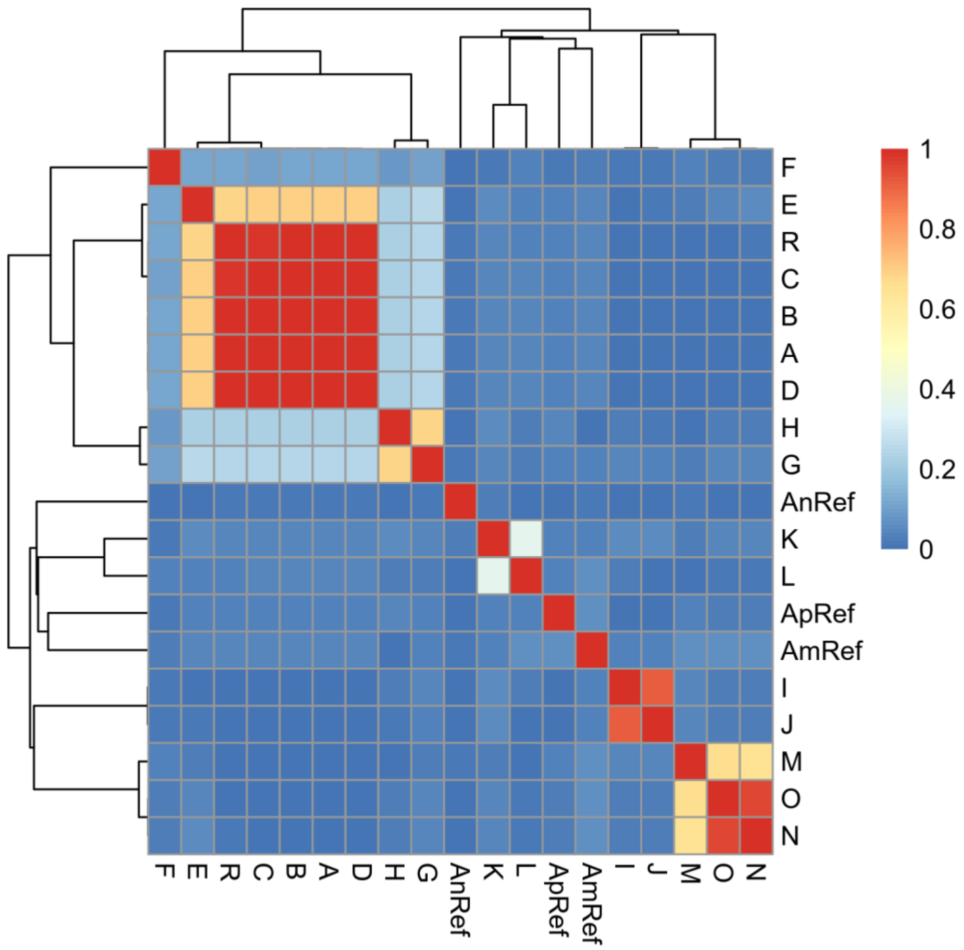

Figure S1: "The intergenomic distances have been calculated with Dashing as described by Gostinčar (2020) using a k-mer lenght 20."
